# Living on the Edge: Using and Improving Trap Crops for Flea Beetle Management in Small-Scale Cropping Systems

**DOI:** 10.3390/insects10090286

**Published:** 2019-09-05

**Authors:** David George, Gordon Port, Rosemary Collier

**Affiliations:** 1 School of Natural and Environmental Sciences, Newcastle University, Newcastle upon Tyne NE1 7RU, UK; 2 Warwick Crop Centre, School of Life Sciences, University of Warwick, Wellesbourne, Warwick CV35 9EF, UK

**Keywords:** trap crop, companion plant, flea beetle, *Phyllotreta*

## Abstract

The use of trap crops to manage pest insects offers an attractive alternative to synthetic pesticides. Trap crops may work particularly well at smaller production scales, being highly amenable where crop diversification and reduction of synthetic inputs are prioritised over yield alone. This paper describes a series of experiments. The first was to demonstrate the potential of turnip rape (*Brassica rapa* L., var. Pasja) as a trap crop to arrest flea beetles (*Phyllotreta* spp.) to protect a main crop of cauliflower (*Brassica oleracea* L., var. Lateman). The subsequent experiments explored two possible approaches to improve the function of the trap crop—either by separating trap and main crop plants spatially, or by introducing companion plants of tomato (*Lycopersicon esculentum* Mill., cv Amateur) into the main crop. In caged field experiments, feeding damage by flea beetles to crop border plantings of turnip rape far exceeded damage to cauliflower plants placed in the same position, indicating a “trap crop effect”. Neither turnip rape plants nor cauliflower as a border significantly reduced flea beetle damage to main crop cauliflower plants, although the numbers of feeding holes in these plants were lowest where a turnip rape border was used. In similar cages, leaving gaps of 3–6 m of bare soil between turnip rape and cauliflower plants significantly reduced feeding damage to the latter, as compared to when plants were adjacent. The results of a small-scale open field trial showed that a turnip rape trap crop alone reduced flea beetle damage to cauliflower, significantly so later in the season at higher pest pressures, but that addition of tomato companion plants did not improve pest control potential.

## 1. Introduction

Farm businesses and subsistence producers at all scales are under increasing pressure to decouple crop production from inputs of synthetic pesticides [1]. Restrictions in pesticide availability and changing political, retailer and consumer demands are encouraging producers to move away from the use of conventional pesticides towards forms of pest management that require fewer synthetic inputs. Approaches to pest management that pose reduced risks to non-target organisms in the environment, avoid issues of pest resistance, promote biodiversity and mitigate consumer exposure to pesticide residues in produce are of particular interest to all stakeholders involved in the food supply chain.

Whilst development of a new generation of (bio)pesticides offers one approach to reducing the use of ‘traditional synthetic pesticides’ [2], inclusion and management of additional flora in the farmed environment could also provide a solution. By substituting external inputs of any kind with additional plant diversity, promotion of “associational resistance” can be achieved [3], either by manipulation of resource concentration effects, or by the promotion of biological control [4]. Increasing plant diversity within crops in this way, for example, through inclusion of companion plants that are not hosts of key herbivorous pest species, has been shown repeatedly to lead to reductions in pest numbers [5,6]. Whilst such approaches may benefit natural enemy populations, particularly if they provide prolonged access to appropriate resources, in annual cropping systems it is generally accepted that ‘companion’ plants more typically interfere with the process of host-plant location and acceptance by pest insects (although there is still some debate as to exactly how these processes are disrupted [7]).

Pest distributions within a given area of crop may also be influenced with trap crops, these being comprised of areas of pest host plants that are positioned near to a main crop to intercept, arrest and retain pests, thereby limiting pest numbers reaching the main crop [8,9]. Trap crops may be especially effective when positioned as borders or strips to intercept pests before they reach the main crop [10]. Dipteran pests, such as cabbage root and carrot fly (*Delia radicum* and *Psila rosae*, respectively), for example, tend to infest crops from field edges due to their reliance on food resources present in these peripheral habitats (e.g., floral nectar/pollen), and/or their natural patterns of infestation [11,12]. For the same reason, trap crops can work well against Lepidoptera, including the diamondback moth, *Plutella xylostella* [9,13,14] and against other groups such as Coleoptera. Predictable, directional (e.g., originating from field edges) movement into crops can be expected, for example, in flea beetles that overwinter in marginal on-farm habitats such as hedgerows. Controlling this key pest group is a current priority in areas of Europe where neonicotinoid seed treatments have recently been banned, and where the resulting inability to effectively manage flea beetles in some crops has led to a substantial reduction in their production (e.g., oilseed rape [15]). Given the ability of these pests to completely decimate seedling brassicas [16], they pose a threat to these crops at both large and small production scales. In larger operations, certain brassicas, such as cauliflower, may be at least partially protected through transplanting as plug-plants at a growth stage that can tolerate feeding. At smaller scales, these species are often sown outdoors from seed, however, exposing the more vulnerable emerging seedling plants to attack.

Trap crops were first suggested as a method of managing flea beetles in 1920, with early plantings of preferred crops recommended as a way of “trapping” adults emerging from hibernation [17]. Later work confirmed the potential for using trap crops to control these pests with, for example, lower numbers of *Phyllotreta cruciferae* recorded in collard crops when these were planted with a border of wild mustard [18]. In later research, a trap crop of radish inter-sown amongst other *Brassica* crop plants was shown to be effective in reducing damage by *Phyllotreta pusilla* in the latter [19] and a trap crop of turnip was employed successfully to protect a main crop of swede from flea beetle attack [20]. Where combinable crops are concerned (oilseed rape), the use of a trap crop of turnip rape has reduced the numbers of adult and larval *Psylliodes chrysocephala* on the main crop [21]. Nevertheless, the level of control achieved by using trap crops to manage pests is variable [8,9] and includes failed attempts to control a range of pests, flea beetles included [22].

Whilst the use of trap crops is being considered in commercial production systems, uptake is historically low, with only ten (successful) examples of trap crops being deployed commercially to 2006 [9], and few new examples since. This may reflect the obvious need to sacrifice land area in order to grow a trap crop, in addition to the logistical, mechanical and agronomic challenges posed by introducing an additional ‘crop’ to a field on a large scale. Such considerations are of reduced concern when cropping at smaller (e.g., subsistence, hobbyist or artisan) scales, however, and as discussed later, trap crops may arguably perform better and be more practically suited to use in these situations. With typically increased flexibility in production approaches at smaller scales, opportunities exist to improve further the effectiveness of trap crops for pest management, for example, by spatially separating them from main crops by small distances (to reduce pest overspill) or by integration with in-crop companion planting. Companion planting itself has a strong history of ‘traditional’ use at hobbyist scales in temperate regions [23] and, when combined with trap crops, the two have been shown to reduce pest damage at (relatively) small scales in African “push-pull” systems [24,25]. For the latter, benefits have not only been demonstrated through control of cereal stemborers (primarily *Chilo partellus* and *Busseola fusca*) on maize, but also through the suppression of striga (a weed) and the promotion of generalist natural enemies such as spiders [26].

This suggests that further research into the use of trap crops, and especially complementary strategies that can be integrated with trap crops, could be of potential use to growers wishing to control pests, and of particular benefit to those producing at smaller scales using more flexible approaches. With this in mind, the aim of the current study was to demonstrate this by: (1) confirming that a trap crop could attract flea beetle feeding damage and protect a main crop, and (2) exploring the potential of spatially separating trap and main crop plants, or introducing companion plants of tomato within the crop, to ascertain if either approach could improve the effectiveness of the trap crop for pest control.

## 2. Materials and Methods

### 2.1. Study Site, Plants and Insects

All experiments were conducted within a walled garden at Close House Field Station, Heddon-on-the-Wall, Northumberland, UK. Based on a previous study [27], cauliflower (*Brassica oleracea* L., var. Lateman) was selected as the main crop, as it “represents a ‘standard’ host plant for these pests” [27], turnip rape (*Brassica rapa* L., var. Pasja) as the trap crop and tomato (*Lycopersicon esculentum* Mill., cv Amateur) as the companion plant.

For two experiments undertaken in cages, all plants used were 5–6 weeks old and had been grown in 9 cm plant pots containing John Innes No. 2 compost in which they remained during the study periods. For the field study, experimental plants were grown in plugs (50 mm^3^) of John Innes No. 2 compost until they were 4.5 weeks old when they were transferred to the field. Further details are provided in Table 1, together with a summary of the environmental conditions during the study period (cage study 1 = 03.08.05–10.08.05; cage study 2 = 01.06.05–14.06.05; field study = 16.06.05–14.08.05).

The composition of the flea beetle population at the study site was assessed by collecting beetles in pitfall traps and on sticky traps. Of the 51 beetles captured during July, the majority were *Phyllotreta* species. Although *Phyllotreta* are only one of twenty genera that comprise the flea beetle populations of Europe, they are considered the most economically significant. Whilst six species of *Phyllotreta* infest brassicas in the UK, by far the most serious as pests are *P. undulata* (L.), *P. nemorum* (L.) and *P. cruciferae* (Goeze), which together made up the bulk of the catch at the study site (41%, 6% and 47% of all individuals trapped, respectively), all of which are common throughout Europe.

### 2.2. Cage Study 1: Is Turnip Rape Effective as a Trap Crop for Flea Beetles?

**Figure 1:**
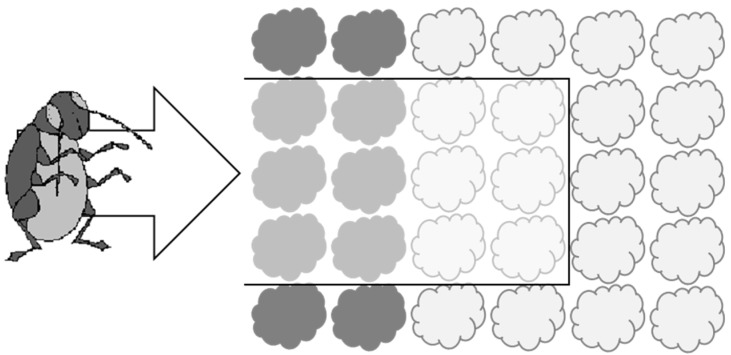
Diagram to show how cages used in Experiment 1 were designed to represent a ‘slice’ through a system incorporating a trap crop (dark shading = border plants) and main crop (light shading = ‘inner’ plants). The cage is shown with a black outline and was open at the front to allow ingress of flea beetles from the ‘field’ edge (direction of travel shown by arrow).

This experiment was conducted using open-fronted cages covered in Enviromesh^®^ netting (‘Ultrafine’ from Agralan) to represent a ‘slice’ through a production system incorporating a trap crop as it might be arranged in the field (see Figure 1). There were three experimental treatments, each consisting of six ‘inner’ main crop cauliflower plants associated with borders of either (i) six turnip rape trap crop plants, (ii) six cauliflower plants or (iii) bare soil (control). The borders were positioned at the front of the cage. The experiment was conducted in early August to coincide with the emergence of the summer generation of adult flea beetles at the study site.

The treatments were arranged in a randomised design, with the position of each treatment allocated at random in each replicate block of three treatments. Two sets of five replicate blocks were aligned in opposite directions. All the cages were 1 m high × 0.6 m wide and were open at the front to allow ingress of flea beetles. The cages were closed at the rear and a grey water trap (24 cm × 37 cm × 5 cm deep) was placed at the far end of each cage to capture any beetles that traversed the full length of the cage. The water traps were approximately 50 cm away from the cauliflower plants at the rear of the cage. The cages were separated from one another by a distance of 50 cm and the whole experimental area was surrounded by at least two rows of potato plants to reduce the effects of any pre-existing bias in the distribution of flea beetles.

The plants were monitored every few days to assess flea beetle feeding damage. When, after 7 days, a measurable level of damage was observed, the plants were removed and stored at 5 °C until the level of damage (feeding holes) could be assessed in the laboratory.

### 2.3. Cage Study 2: Can the Effectiveness of Trap Crops Be Improved by Spatial Separation From Main Crops?

**Figure 2:**
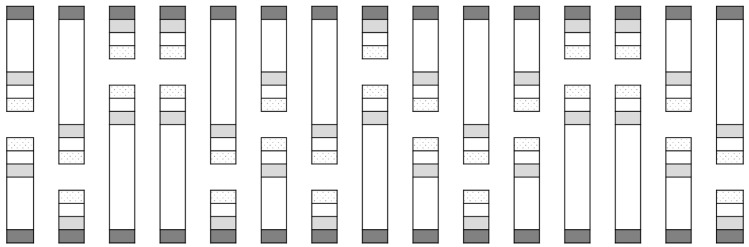
Layout of field cages for Cage Study 2. The areas shaded in dark grey represent turnip rape border plants placed at cage openings. The areas shaded in light grey represent cauliflower plants inside cages at varying distances from the turnip rape plants. The un-shaded checkered areas at the very end of the cages indicate the location of water traps.

This experiment was similar to the first and was run using similar open-fronted cages. There were ten replicates of three treatments, which were run concurrently in early June of the same year as Cage Study 1 (to coincide with emergence of overwintered flea beetles at the study site). A border of six turnip rape plants was placed at the front of each cage at distances of 0, 3 or 6 m from six ‘inner’ cauliflower plants. The treatments were arranged in a randomised design, with the position of each treatment allocated at random in each replicate block of three treatments. Two sets of five replicate blocks were aligned in opposite directions (Figure 2). The cages differed in length according to treatment, being 1-m longer than the treatment they contained (i.e., 1 m for the 0 m treatment, 4 m for the 3 m treatment and 7 m for the 6 m treatment), with all other dimensions as per Cage Study 1.

The plants were assessed for damage every few days. When, after 14 days, a measurable level of damage was observed, plants were removed and stored at 5 °C until the numbers of feeding holes could be recorded in the laboratory.

### 2.4. Field Study: Can the Effectiveness of Trap Crops be Improved Through Combination with Companion Plants?

**Figure 3:**
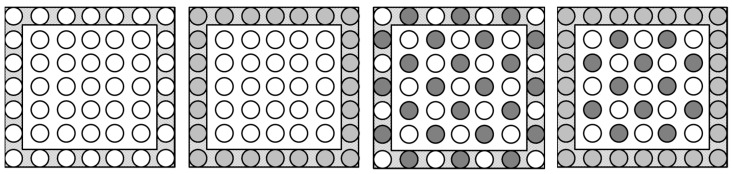
Layout of treatments used in the field study. One circle = one plant, where border and ‘inner’ plants are presented in a shaded (grey) and un-shaded (white) background, respectively. Key to circles/plants: white = cauliflower plant; light grey = turnip rape; dark grey = tomato.

Four treatments were chosen to represent the way in which trap crops and companion plants are commonly deployed in the field. The treatments were as follows (Figure 3): (i) cauliflower monoculture (49 plants in a 7 × 7 block, with this same spatial arrangement used for all treatments); (ii) cauliflower (25 plants) with a trap crop border (turnip rape) (24 plants); (iii) cauliflower (25 plants) with companion plants (tomato) (24 plants, inter-cropped); (iv) cauliflower (13 plants) and companion plants (tomato) (12 plants, inter-cropped) with a trap crop border (turnip rape) (24 plants). These treatments were set out as a randomised block design with three replicates of each treatment.

Each plant was set in its own 25 cm × 25 cm space within a 7 × 7 (i.e., 175 cm × 175 cm) planted plot, and a gap of 2 m of bare soil surrounded any plot. To aid establishment, plants were watered as necessary for the first three weeks after transplanting. After this the plants were well-established and required no further artificial irrigation. Gaps between plants and replicates were kept weed-free by a combination of rotovation and manual weeding.

In each treatment, eight border (turnip rape or cauliflower) plants were selected at random for assessment, as were eight ‘inner’ cauliflower plants. Feeding damage due to flea beetles (holes in leaves) was assessed in situ on all selected host plants on 19, 22, 25 June and 15 July, 3, 6, 9 and 29 days after transplanting (DAT), respectively. Damage to the ‘inner’ cauliflower plants was also assessed on 4 August (49 DAT), though damage to border plants was not assessed on this date as it was difficult to reliably count individual shot holes in turnip rape at this time (due to their sheer number). Although the cauliflower plants were still immature at the end of the experiment, they had started to produce curds and would have been at point of harvest shortly after the final assessment. 

### 2.5. Data Analysis

For Cage Study 1, the effect of treatment on feeding damage to the border plants (turnip rape or cauliflower), the ‘inner’ cauliflower plants, and the two sets of plants combined, was analysed by a two-way ANOVA (with replication), considering treatment and cage aspect as factors and examining the interaction between them. Data on damage to border plants alone and ‘all plants’ were log transformed prior to analysis in order to ensure homogeneity of variances. Data for percentage feeding preference for border plants, vs ‘inner’ cauliflower, in the two treatments that contained them were also analysed (i.e., the percentage of feeding holes recorded in the border plants as a function of all feeding holes recorded in both border and ‘inner’ plants). These data could not be made to fit the assumptions for parametric testing and so they were analysed using a Mann–Whitney Test. Paired T-tests were used to assess feeding preference for border vs ‘inner’ plants within treatments, where data again required log transformation prior to analysis. Where initial analysis by ANOVA identified statistically significant effects, Tukey’s Tests were performed to identify differences between pairs of means. In this and subsequent analyses, Kolmogorov-Smirnov and Levenes Tests were used to assess normality and homoscedasticity of variances, respectively, with all analyses being run using SPSS v. 17.

For Cage Study 2, the effect of treatment on feeding damage to the turnip rape plants, the ‘inner’ cauliflower plants, the two combined, and the percentage feeding preference for the turnip rape (vs the cauliflower) between treatments (calculated as in the previous experiment) were analysed using a two-way ANOVA (with replication) considering treatment and cage aspect as factors and examining the interaction between them. Data for the ‘inner’ cauliflower plants were log transformed prior to analysis in order to ensure homogeneity of variances, with the percentage feeding preference data requiring arcsin square root transformation for the same reason. Paired t-tests were used to assess feeding preferences for border (turnip rape) vs ‘inner’ (cauliflower) plants within the treatments, where data again required log transformation prior to analysis. Where initial analysis by ANOVA identified statistically significant effects, Tukey’s Tests were performed to identify differences between pairs of means.

For the Field Study, data from the first assessment (3 DAT) were not analysed as levels of flea beetle damage were negligible at that time. Data on feeding damage to ‘inner’ cauliflower plants was analysed using 2-way ANOVA (without replication), considering treatment and block as main factors and analysing data from different sampling dates (DAT) independently. Data for feeding damage to border plants (log transformed), and ‘inner’ and border plants combined (log transformed), were analysed in the same way, as were data for percentage feeding preference for the border plants between treatments (arcsin square root transformed), with percentage feeding preference calculated as in previous experiments. Paired *t*-tests were used to assess feeding preference for border vs ‘inner’ plants within the treatments on each sampling date, where data were again log transformed prior to analysis. Where ANOVA identified statistically significant differences between treatments, the Tukey Test was used to identify differences between pairs of means.

## 3. Results

### 3.1. Cage Study 1: Is Turnip Rape Effective as a Trap Crop for Flea Beetles?

**Figure 4:**
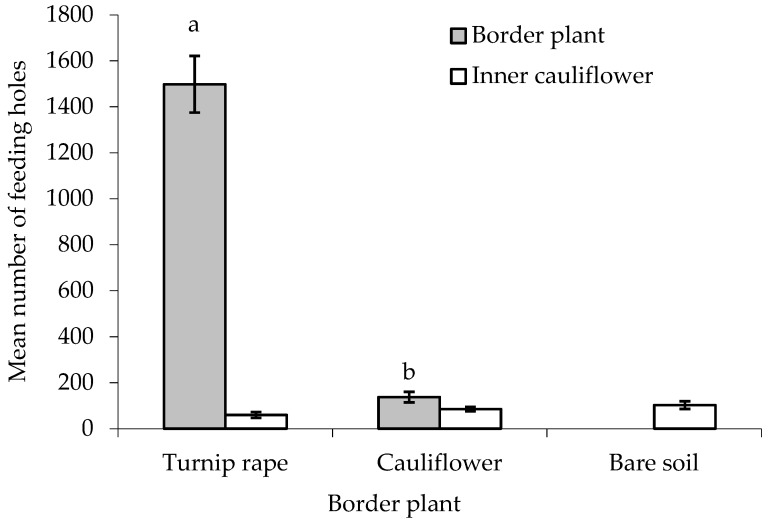
Number of flea beetle feeding holes (mean ± SE) on leaves of turnip rape or cauliflower plants used for the border and in ‘inner’ cauliflower plants under different treatments. n = 10 for all means. Means with different letters (a–b) denote statistically significant differences between treatments.

There was no statistically significant difference between treatments in the number of feeding holes made by flea beetles in the leaves of the ‘inner’ main crop cauliflower plants, although a *p*-value approaching significance was obtained (F_(2,24)_ = 2.581, *p* = 0.097, cage aspect and interaction with treatment not significant) (Figure 4). When comparing the two treatments with border plants, both turnip rape and cauliflower plants in the border suffered significantly higher levels of feeding damage than the cauliflower they were ‘protecting’ (T_(9)_ = 27.79 and 2.78, where *p* < 0.001 and 0.05, respectively). More feeding holes were, however, made in the turnip rape plants than the cauliflower plants in the border (F_(1,16)_ = 207.19, *p* < 0.001, cage and interaction with treatment aspect not significant).

There were statistically significant differences between treatments for both the total number of feeding holes and the percentage feeding preference for the different border plant species (Table 2). Total feeding holes were highest in the treatment containing turnip rape plants and lowest where no border plants were included, with percentage feeding preference for the border plants significantly higher where these were turnip rape as opposed to cauliflower.

### 3.2. Cage Study 2: Can the Effectiveness of Trap Crops Be Improved by Spatial Separation from Main Crops?

**Figure 5:**
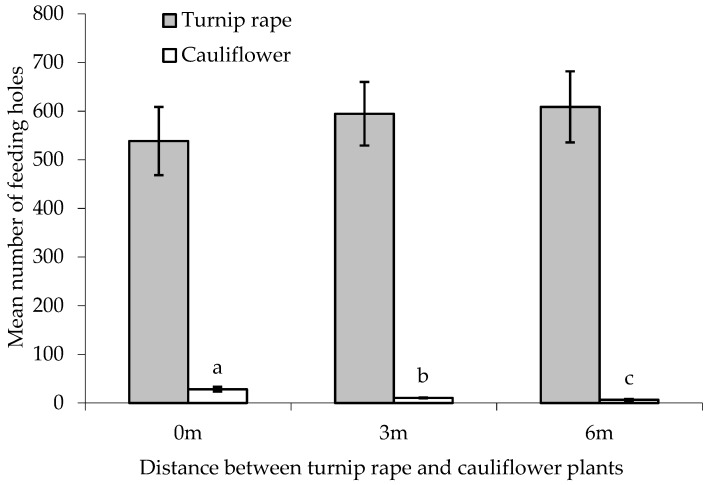
Number of flea beetle feeding holes (mean ± SE) on leaves of cauliflower plants and turnip rape plants separated by different distances. n = 10 for all means. Means with different letters (a–c) denote significant differences between treatments.

There was a statistically significant difference between treatments in the number of feeding holes on the ‘inner’ main crop cauliflower plants (F_(2,24)_ = 18.69, *p* < 0.001, cage aspect and interaction with treatment not significant). Progressively fewer holes were found on ‘inner’ cauliflower plants with increasing distance from the turnip rape border plants (Figure 5). In all treatments (0, 3 and 6 m separation between turnip rape and cauliflower plants), significantly more holes were found on the turnip rape plants than on the cauliflower (T_(9)_ = 14.86, 33.41 and 17.54, respectively, *p*(2-tailed) < 0.001 in all cases). Feeding damage to turnip rape plants did not differ significantly between treatments (F_(2,24)_ = 0.46, *p* = 0.636, cage aspect and interaction with treatment not significant).

There was no statistically significant difference between treatments in the total number of feeding holes recorded, though feeding preference for turnip rape border plants was affected by treatment (Table 3), being lower where these plants were adjacent to ‘inner’ cauliflower.

### 3.3. Field Study: Can the Effectiveness of Trap Crops Be Improved through Combination with Companion Plants?

**Figure 6:**
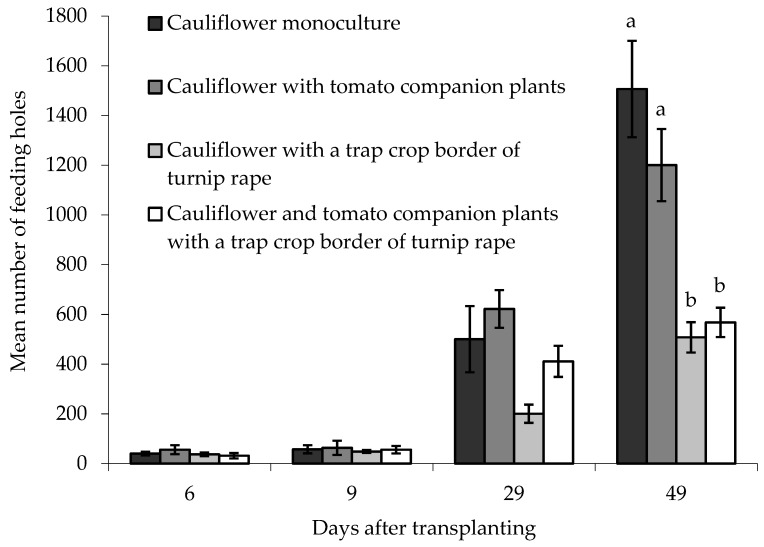
Number of flea beetle feeding holes (mean ± SE) on leaves of eight ‘inner’ cauliflower plants grown under different treatments at different days after transplanting. n = 3 for all means. Means with different letters (a–b) denote significant differences between treatments.

On the ‘inner’ main crop cauliflower plants, there was no statistically significant difference between treatments in the numbers of feeding holes when plants were sampled 6, 9 or 29 DAT (Figure 6). There was, however, a significant difference between treatments 49 DAT (F_(3,6)_ = 16.68, *p* < 0.01) where more feeding holes were observed in ‘inner’ cauliflower plants grown either as a monoculture or with tomato companion plants, compared with cauliflower grown with a turnip rape trap crop, or with a turnip rape trap crop and companion tomato plants together. Block did not have a statistically significant effect on the data.

**Figure 7:**
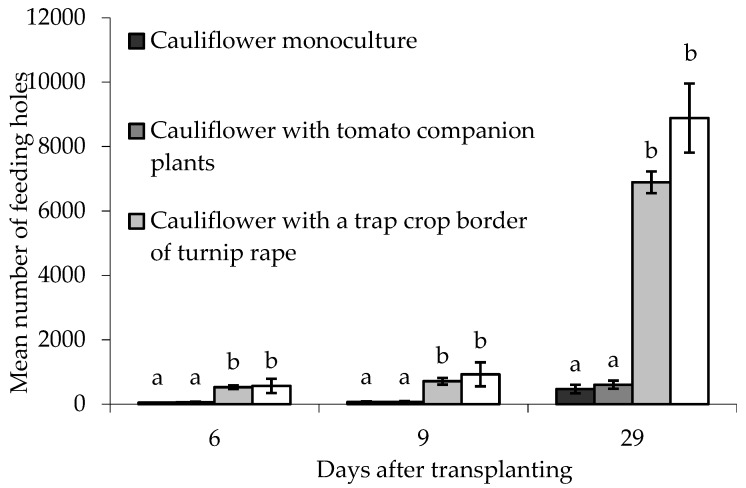
Number of flea beetle feeding holes (mean ± SE) on leaves of eight border plants grown under different treatments at different times after transplanting. n = 3 for all means. Means with different letters (a–b) denote significant differences between treatments on any given sampling occasion.

Feeding holes in border plants were assessed 6, 9 and 29 DAT. There were statistically significant differences between treatments on all sampling dates (6 DAT; F_(3,6)_ = 25.32, *p* < 0.01, 9 DAT; F_(3,6)_ = 18.68, *p* < 0.01, 29 DAT; F_(3,6)_ = 47.56, *p* < 0.001). In all cases there were significantly more holes in the turnip rape plants (both treatments) than in similarly situated cauliflower plants in treatments without turnip rape (Figure 7). Block did not significantly affect the data on any sampling date.

In the case of the treatments with cauliflower alone or with only companion plants (tomato), the numbers of feeding holes on cauliflower plants in the border were not significantly different to those on ‘inner’ main crop cauliflower plants on any sampling date. Conversely, for both the treatments using turnip rape trap crop plants, significantly more holes were recorded on turnip rape plants in the border than the ‘inner’ cauliflower plants (for 6, 9 and 29 DAT, respectively: turnip rape trap crop alone (T_(2)_ = −21.260; −10.095; −24.004, *p*(2-tailed) < 0.01; = 0.01; < 0.01; turnip rape and tomato companion plants (T_(2)_ = −21.816; −6.036; −34.896, *p*(2-tailed) < 0.01; < 0.05; = 0.001).

When considering the total number of holes made on all plants of a single treatment, or the percentage feeding preference for border plants, there were statistically significant differences between treatments on all sampling dates (Table 4). In all cases, this was because there were significantly more holes on, or an increased preference for, border plants in treatments containing turnip rape compared with those without. Pair-wise differences were non-significant only in the case of percentage feeding preference data collected 9DAT. Block did not significantly affect these data on any sampling date.

## 4. Discussion

### 4.1. Cage Study 1: Is Turnip Rape Effective as a Trap Crop for Flea Beetles?

Although there was no statistically significant difference in the numbers of feeding holes on ‘inner’ main crop cauliflower plants ‘protected’ with a border of turnip rape vs those without a border, the presence of the turnip rape did lead to a ca. 40% reduction in feeding damage. Though some studies have suggested that the use of trap crops may not work especially well for flea beetles [22], host preference hierarchies have been demonstrated in this group [28] and the balance of published research suggests that the use of trap crops has potential for the management of flea beetles [17,18,19,20,29], including the use of turnip rape [21]. A border of cauliflower plants was also ineffective in reducing the number of feeding holes on the ‘inner’ cauliflower plants, with an even smaller reduction in feeding damage (ca. 22%) compared with the absence of border plants. Although not statistically significant, this trend suggests that an interception, or edge effect reduced pest damage on the ‘inner’ cauliflower plants, with this being greater when a preferred host plant (turnip rape) was used as a trap crop. Evidence for such an edge effect per se was demonstrated by the significantly higher damage on border vs ‘inner’ plants, with this effect being stronger where turnip rape was used. 

Reliance on an initial edge effect (i.e., to intercept pests on route to a main crop) may explain why trap crops have been relatively ineffective for the management of pests that move into a crop in a largely undirected manner [8,9]. Attempts to model the effect of trap crops on pest insects support the view that undirected/random dispersers are unlikely to be controlled efficiently through this approach [30]. Though exceptions occur (e.g., [19]), the often-reported failure of inter-planted trap crops to reduce pest damage could also be attributed to a loss of edge effects. Inter-planted Chinese cabbage trap crops, for example, did not control *Phyllotreta* spp. in white cabbage [22], and though a trap crop of sweet alyssum reduced damage by *P. xylostella* to cabbage when planted as a border around it, it had no effect when the same species was inter-cropped with cabbage [31].

### 4.2. Cage Study 2: Can the Effectiveness of Trap Crops Be Improved by Spatial Separation from Main Crops?

Leaving areas of bare soil between the turnip rape and main crop ‘inner’ cauliflower plants did not affect the overall amount of feeding in any treatment but reduced the incidence of flea beetle damage on the latter, with the use of increasing distances being more effective. When adjacent to each other, recognition of, and movement to, main crop plants from trap crop plants is likely to be far easier for pest insects than when the two are spatially separated and could even occur ‘accidentally’ during bouts of exploratory behaviour or trivial flights. Such behaviour has been studied in detail for the cabbage root fly, *Delia radicum* [32], and described for *Phyllotreta* spp. [33], where beetle numbers may be relatively high on main crop plants adjacent to trap crops [20]. This overspill of pests from a trap crop to a main crop has been cited as a key reason for trap crop failure in commercial settings, with methods to improve pest retention on trap crops identified as being crucial to overcoming this limitation [34].

Despite the apparent effectiveness of the areas of bare soil between turnip rape borders and the cauliflower plants, it is unlikely that pest management approaches that involve leaving large tracts of soil bare would be adopted readily in commercial horticulture. In smaller holdings, however, it may be possible to take advantage of existing barriers, such as access routes to farms and allotments, to provide these ‘barren’ tracts, particularly if their widths need only be a few meters to exert a positive effect, as suggested here. Such features could also be utilised at larger scales but are less likely to be adjacent to cropped areas in more extensive production systems.

At all production scales, less space-intensive pest management techniques could have a similar effect to spatially separating trap and main crops. The use of border fences around the main crop, for example, has been investigated [35,36] and could provide a comparable barrier. Alternatively, trap crops could be separated from main crops by being alongside agri-environment measures, such as sown field margins, or their equivalents in hobbyist production settings (e.g., floral borders in a garden or grassy verges and hedge lines on allotments). This could be a particularly attractive option for pest management, making optimum use of natural enemies concentrated in these habitats to manage pests aggregated within easy reach on adjacent trap crops [11].

### 4.3. Field Study: Can the Effectiveness of Trap Crops Be Improved through Combination with Companion Plants?

In the early part of the field study, feeding damage by flea beetles was low and (statistically) similar in all treatments. As damage increased, however, differences between treatments were more apparent and confirmed the potential for a border trap crop of turnip rape to reduce flea beetle damage to a main crop of cauliflower (in both treatments that included a trap crop). A similar temporal trend has been observed for *Lygus* spp. on lettuce, where a trap crop of alfalfa had no effect on pest numbers until approximately one month into the trial [37]. The potential for trap crops to attract relatively high levels of pest damage was also evident in this experiment, with significant preferences for the turnip rape plants driving significantly more flea beetle feeding on the borders, and consequently increased feeding overall, in plots containing trap crop plants. Whilst this suggests a risk of flea beetle overspill from trap crops in these plots, this did not appear to occur over the duration of this field study, which spanned almost the entire growing season of the (main) crop. This could be attributed, in part, to selection of a large and robust species as a trap crop that was able to tolerate high levels of flea beetle damage whilst remaining attractive as a host.

Even with the relatively effective trap crop treatment(s) used here, any reduction in feeding damage to the ‘inner’ cauliflower crop was at the cost of reducing the number of cauliflower plants grown in the plot by a minimum of ca. 50%. Trap crops grown at a commercial scale typically occupy lower percentages of the total field area, 10% being the ‘norm’ [8], although even at this level, removing land from main-crop production and ensuring that main and trap crops can be established and managed in a single field present barriers to commercial uptake. At smaller scales, however, growers are arguably more likely to prioritise the reduction of synthetic inputs above yield, particularly in artisan, organic and hobbyist systems where produce is either grown for self-consumption or marketed at a premium based on minimal or zero pesticide use. At these smaller scales, growers are also less likely to need to invest in additional machinery and agronomy to co-establish main and trap crops in a single field (as achieving 10% cover will only involve a relatively limited number of plants). At these same smaller scales, growers, and especially subsistence farmers, are also more likely to view the trap crop as a commodity in its own right, as well as assigning added value to any other services it could deliver. Trap crops in the aforementioned African ‘push-pull’ systems, for example, are often harvested as fodder [24,38], and trap crops, per se, may bolster the numbers of natural enemies [39] and provide floral resources for pollinators—without disrupting main crop pollination in systems where this is required [40]. Turnip rape ‘Pasja’, as used in the current study, is ideal fodder for all grades of cattle stock and may be highly productive, with a rapid growth habit that can yield over four tons of dry matter per acre [41].

The use of companion plants may be similarly disproportionally attractive to small vs large scale production, for much the same reasons as trap crops. Tomato plants used as companions had no effect on flea beetle damage to cauliflower in the current study, however, and (unsurprisingly then) did not improve the effectiveness of trap crops when combined with a border of turnip rape. Consequently, this work supports the suggestion that, at least at smaller scales, using trap crops might hold more promise than other forms of polyculture for managing pest populations [42]. For flea beetles, it may even be the case that gains to trap crop performance would be better achieved by diversifying the trap crop itself rather than the main crop stand, i.e., by including multiple species within the trap crop to improve its pest control potential [29]. Nevertheless, polyculture per se has been employed with success against flea beetles elsewhere. Early studies by Andow et al. [43], where cabbage was inter-planted with a range of living mulches (all non-host plants for pest species), demonstrated that the numbers of *Phyllotreta* spp. could be reduced by polyculture. These observations were confirmed later by other researchers using cabbage under-sown with clover [44], a mixed planting of faba bean or vetch in broccoli [45], when intercropping Chinese cabbage with onion [46], when inter-planting basil and Chinese kale [47], and when white cabbage was intercropped with marigold [48], a species with a strong traditional history of use as a companion plant in gardens and allotments [23].

It is possible that companion plants may have been more successful in the current study if a higher ratio of companion to main crop plants had been used, as was the case in related work with flea beetles that led to selection of tomato as the companion species here [27]. In support of this suggestion, several other studies have shown non-host companion plants, and indeed plant models, to deliver more effective control of other *Brassica* pests when deployed at higher densities and/or with greater leaf areas (re *D. radicum* [49,50], *Delia antiqua* [49] and *P. xylostella* [51]). In this study, companion plants were also used in place of cauliflower plants in a substitutive design, rather than being used additively. It could therefore be argued that the presence of companion plants may have reduced the overall number of flea beetles in a plot, but that damage to individual ‘inner’ main crop cauliflower plants remained relatively consistent across treatments (i.e., because there were fewer of these cauliflower plants where companion plants were used). Thus, whilst it appeared in this study that there was no benefit in using either companion plants alone or in conjunction with a turnip rape border for flea beetle control, this conclusion can only be drawn tentatively.

## 5. Conclusions

In conclusion, using a border of turnip rape as a trap crop reduced flea beetle feeding damage to small plots of cauliflower plants. Companion planting with tomato did not improve the effectiveness of a trap crop border of turnip rape to protect cauliflower from flea beetle attack, although as the use of tomato as a companion plant alone was ineffective, this was not unexpected. Results suggest that barriers to dispersal from the trap crop to main crop plants may present a more efficient means of improving trap crop effectiveness at small scales, where it is at these scales that trap crops are arguably more complementary to crop production from both a practical and functional perspective.

## Figures and Tables

**Table 1:** Plant growth characteristics and summary of environmental conditions for the three experiments conducted. All plant measurements were taken at the end of experiments. ht = height; no. = number; n = number of plants measured; min = minimum; max = maximum; temp = temperature. Leaf areas were assessed using a Delta T bench-top leaf area meter.

		Plant Growth Characteristics				Environmental Conditions			
Experiment	Plant Species	Mean ht (cm)	Mean Leaf no.	Mean Leaf Area (cm^2^ × 0.01)	n	Mean Min Temp (°C)	Mean Max Temp (°C)	Mean Daily Rainfall (mm)	% Days Rain Free
Cage study 1	Cauliflower	24.4 ± 0.4	7.7 ± 0.2	29.6 ± 0.8	10	9.6 ± 0.8	17.1 ± 1.4	1.4 ± 0.6	50
	Turnip rape	36.9 ± 1.4	9.8 ± 0.4	91.3 ± 9.0	10				
Cage study 2	Cauliflower	26.5 ± 0.3	8.9 ± 0.1	48.3 ± 1.4	30	8.7 ± 0.7	15.1 ± 0.8	1.4 ± 0.5	50
	Turnip rape	23.6 ± 0.5	10.3 ± 0.3	62.8 ± 2.9	30				
Field study	Cauliflower	30.0 ± 0.8	11.0 ± 0.3		96	11.5 ± 0.3	18.5 ± 0.5	2.8 ± 0.7	54
	Turnip rape	65.9 ± 1.9	39.3 ± 2.0		24				
	Tomato	60.0 ± 1.9	72.2 ± 3.4		24				

**Table 2:** Mean percentage feeding preference for border plants and total number of feeding holes on both border and ‘inner’ plants. Means are displayed with ± SEs. n = 10 for all means. Means with different letters (a–c) denote significant differences between treatments within a column.

Border Plant Treatment	% Preference for Border Plants	Total Number of Feeding Holes
Turnip rape	96.41 ± 5.22a	1557.50 ± 133.58a
Cauliflower	60.39 ± 3.66b	218.80 ± 26.50b
Bare soil	NA	102.70 ± 16.81c
Z/F_(2,24)_	−3.78	108.59
*p*	<0.001	<0.001

**Table 3:** Mean percentage feeding preference for border plants and total number of feeding holes in both border and ‘inner’ plants. Means are displayed with ± SEs. n = 10 for all means. Means with different letters (a–b) denote significant differences between treatments within a column.

Treatment	% Preference for Border Plants	Total Number of Feeding Holes
0 m	94.30 ± 0.96a	538.60 ± 70.17
3 m	98.11 ± 0.22b	594.60 ± 65.45
6 m	98.75 ± 0.37b	608.80 ± 73.10
F_(2,24)_	18.82	0.26
*p*	<0.001	0.77

**Table 4:** Mean percentage feeding preference for border plants and total number of feeding holes in both border and ‘inner’ plants. Means are displayed with ± SEs. n = 3 for all means. Means with different letters (a–b) denote significant differences between treatments within a column.

	% Preference for Border Plants			Total Number of Feeding Holes		
Treatment	6 DAT	9 DAT	29 DAT	6 DAT	9 DAT	29 DAT
Cauliflower monoculture	55.20 ± 4.37a	55.03 ± 11.29	47.99 ± 0.67a	88.67 ± 9.06a	125.00 ± 16.80a	967.67 ± 265.06a
Cauliflower with tomato companion plants	52.62 ± 13.70a	53.18 ± 13.89	48.33 ± 3.20a	116.00 ± 11.37a	132.00 ± 33.62a	1224.33 ± 196.46a
Cauliflower with a trap crop border of turnip rape	93.51 ± 0.72b	93.27 ± 1.45	97.19 ± 0.43b	561.33 ± 64.28b	759.67 ± 99.35b	7091.33 ± 359.34b
Cauliflower and tomato companion plants with a trap crop border of turnip rape	94.54 ± 0.70b	92.84 ± 2.97	95.57 ± 0.36b	598.33 ± 232.98b	981.00 ± 370.95b	9294.67 ± 1123.06b
F_(3,6)_	14.17	6.15	302.38	20.48	16.41	33.57
*p*	0.004	0.29	<0.001	0.001	0.003	<0.001
